# A Universal Random Coding Ensemble for Sample-Wise Lossy Compression

**DOI:** 10.3390/e25081199

**Published:** 2023-08-11

**Authors:** Neri Merhav

**Affiliations:** The Viterbi Faculty of Electrical and Computer Engineering, Technion—Israel Institute of Technology, Technion City, Haifa 3200003, Israel; merhav@ee.technion.ac.il; Tel.: +97-248-294-737

**Keywords:** rate–distortion, source coding, random coding, cumulant generating function, code ensemble, lossy compression, universal coding, universal distribution, LZ algorithm

## Abstract

We propose a universal ensemble for the random selection of rate–distortion codes which is asymptotically optimal in a sample-wise sense. According to this ensemble, each reproduction vector, $\hat{x}$, is selected independently at random under the probability distribution that is proportional to $2^{-LZ(\hat{x})}$, where $LZ(\hat{x})$ is the code length of $\hat{x}$ pertaining to the 1978 version of the Lempel–Ziv (LZ) algorithm. We show that, with high probability, the resulting codebook gives rise to an asymptotically optimal variable-rate lossy compression scheme under an arbitrary distortion measure, in the sense that a matching converse theorem also holds. According to the converse theorem, even if the decoder knew the *ℓ*-th order type of source vector in advance (*ℓ* being a large but fixed positive integer), the performance of the above-mentioned code could not have been improved essentially for the vast majority of codewords pertaining to source vectors in the same type. Finally, we present a discussion of our results, which includes among other things, a clear indication that our coding scheme outperforms the one that selects the reproduction vector with the shortest LZ code length among all vectors that are within the allowed distortion from the source vector.

## 1. Introduction

We revisit the well-known problem of lossy source coding for finite-alphabet sequences with respect to a certain distortion measure ([1]; [2], Chapter 10; [3], Chapter 9; [4]; [5], Chapters 7 and 8). More concretely, our focus is on *d*-semifaithful codes, namely, variable–rate codes that meet a certain distortion constraint for every source sequence (and not only in expectation). As is very well known [1], the rate–distortion function quantifies the minimum achievable expected coding rate for a given memoryless source and distortion measure.

During several past decades, many research efforts were motivated by the fact that the source statistics are rarely (if not never) known in practice, and were, therefore, directed to the quest for universal coding schemes, namely, coding schemes which do not depend on the unknown statistics, but nevertheless, approach the lower bound (i.e., the entropy in lossless compression, or the rate–distortion function in the lossy case) asymptotically, as the block length grows without bound. We next provide a very brief (and non-comprehensive) review of some of the relevant earlier work.

In lossless compression, the theory of universal source coding is very well developed and mature. Davisson’s work [6] concerning universal coding redundancies has established the concepts of weak universality and strong universality (vanishing maximin and minimax redundancies, respectively), and has characterized the connection to the capacity of the ‘channel’ defined by the family of conditional distributions of the data to be compressed given the index (or parameter) of the source in the class [7,8,9]. For many of the frequently encountered parametric classes of sources, the minimum achievable redundancy of universal codes is well known to be dominated by $\frac{k\log n}{2n}$, where *k* is the number of degrees of freedom of the parameter, and *n* is the block length [10,11,12,13]. A central idea that arises from Davisson’s theory is to construct a Shannon code pertaining to the probability distribution of the data vector with respect to a mixture (with a certain prior function) of all sources in the class. Rissanen, who was the inventor of the minimum description length (MDL) principle [14], has proved in [15] a converse to a coding theorem, which asserts that asymptotically, no universal code can achieve redundancy below $\left(1-\epsilon \right)\frac{k\log n}{2n}$, with the possible exception of sources from a subset of the parameter space, whose volume tends to zero as n→∞ for every positive ϵ. Merhav and Feder [16] have generalized this result to more general classes of sources, with the term $\frac{k\log n}{2n}$ substituted by the capacity of the above-mentioned ‘channel’. Further developments, including more refined redundancy analyses, have been carried out in later studies.

In the wider realm of universal lossy compression, the theory is, unfortunately, not as sharp and well developed as in the lossless setting. We confine our attention, in this work, to *d*-semifaithful codes [17], namely, codes that satisfy the distortion requirement with probability one. Zhang, Yang, and Wei [18] have proved that, unlike in lossless compression, in the lossy case, even if the source statistics are known perfectly, it is impossible to achieve redundancy below $\frac{\log n}{2n}$, but $\frac{\log n}{n}$ is achievable. Not knowing the source conveys the price of enlarging the multiplicative constant in front of $\frac{\log n}{n}$. Indeed, Yu and Speed [19] have established weak universality with a constant that grows with the cardinalities of the alphabets of the source and the reconstruction [20]. Ornstein and Shields [17] have considered universal *d*-semifaithful coding for stationary and ergodic sources with respect to the Hamming distortion measure, and established convergence with probability one to the rate–distortion function. Kontoyiannis [21] had several interesting findings. The first is a certain central limit theorem (CLT), with an $O(1/\sqrt{n})$ redundancy term, whose coefficient is described as a limiting Gaussian random variable with some constant variance. The second is the so-called law of iterated logarithm (LIL), with redundancy proportional to $\sqrt{\frac{\log(\log n)}{n}}$ infinitely often with probability one. One of the counter-intuitive conclusions from [21] is that universality is priceless under those CLT and LIL criteria. In [22], many of the findings are based on the observation that optimal compression can be characterized in terms of the negative logarithm of the probability of a sphere of radius nD around the source vector with respect to the distortion measure, where *D* is the allowed per-letter distortion. In the same article, they proposed also the ensemble of random coding with respect to a probability distribution given by a mixture of all distributions in a certain class. In two recent articles, Mahmood and Wagner [23,24] have studied *d*-semifaithful codes that are strongly universal with respect to both the source and the distortion function. The redundancy rates in [23] behave like $\frac{\log n}{n}$ with different multiplicative constants. Other interesting results concerning a special distortion measure appear in [25].

A parallel line of research work on universal lossless and lossy compression, which was pioneered by Ziv, pertains to the individual sequence approach. According to this approach, there are no assumptions on the statistical properties of the source. The source sequence to be compressed is considered an arbitrary deterministic (individual) sequence, but limitations are imposed on the encoder and/or the decoder to be implementable by finite-state machines. This includes, first and foremost, the celebrated Lempel–Ziv (LZ) algorithm [26,27,28], as well as further developments that extend the scope to lossy compression with and without side information [29,30], as well as to joint source–channel coding [31,32]. In the lossless case, the article [33] provides an individual sequence analogue of the above-mentioned result due to Rissanen, where the expression $\frac{k\log n}{2n}$ continues to designate the best achievable redundancy, but the main term of the compression ratio there is the empirical entropy of the source vector instead of the ordinary entropy of the probabilistic setting. The converse bound of [33] applies to the vast majority of source sequences within each type, and the vast majority of types (in analogy to the vast majority of the parameter space in Rissanen’s framework). In a way, this kind of a converse result still contains some flavor of the probabilistic setting, because arguing that the number of exceptional typical sequences is relatively small is actually equivalent to imposing a uniform distribution across the type and asserting that the induced probability of violating the bound is small. The achievability result of [33], on the other hand, holds pointwise for every sequence. A similar comment applies to [34], where asymptotically pointwise lossy compression was established with respect to first-order statistics (i.e., “memoryless” statistics) with an emphasis on distortion universality, similar to in [23,24].

A similar kind of a mix between the individual sequence setting and the probabilistic setting applies to this paper as well, in the context of universal rate–distortion coding, but here, just like in [34], there is no limitation to finite-state encoders/decoders as in [33]. In particular, our converse theorem asserts that given an arbitrary variable-rate code, and given an arbitrary distortion function within a certain wide class, the vast *majority* of reproduction vectors that represent source sequences of a given type (of any fixed order), must have a code length that is essentially at least as large as the negative logarithm of the probability of a ball with normalized radius *D* (*D* being the allowed per-letter distortion) centered at the given source sequence. The probability of this ball is taken with respect to a universal distribution that is proportional to $2^{-LZ(\hat{x})}$, where $LZ(\hat{x})$ is the code length of LZ encoding of the reproduction vector, $\hat{x}$.

The emphasis on the word “majority” in the previous paragraph (which was also mentioned earlier) calls for an explanation: it should be understood that in the absence of limitations on the encoding memory resources (such as the finite-state machine model described above), there cannot exist any non-trivial lower bound that applies to each and every given individual sequence. The reason is simple: given a particular individual source sequence, one can always tailor an encoder that compresses that sequence to a single bit (even losslessly), for example, by assigning the bit ‘0’ to be the compressed representation of the given sequence and by appending the bit ‘1’ as a header to the uncompressed binary representation of any other source sequence. For the given individual sequence, the compression ratio would then be 1/n, which tends to zero as *n* grows without bound (of course, this encoder would be very poor for many other source sequences). It is, therefore, unavoidable that any non-trivial lower bound must apply collectively over some set of source sequences, allowing at least some minority of exceptional sequences that may violate the bound.

We also present a matching achievability result, asserting that for every source sequence, this code length is essentially achievable by random coding, using a universal ensemble of codes, which is defined by independent random selection, where each codeword is drawn under the above-described universal probability distribution.

While the achievability result in [34] was pointwise as well, it was tailored to a memoryless structure, in the sense that it was given in terms of the rate–distortion function of the first-order empirical distribution, which is blind to any empirical dependencies and repetitive patterns within the source sequence. In this paper, we both extend the scope to general individual sequences beyond the memoryless statistics and extend the allowable class of distortion measures. In terms of the technical aspects, the proof of the achievability result is similar to a parallel proof in [34], but the novelty lies considerably more in the converse theorem and its proof, which is very different from the one in [34] because here there is no information-theoretic single-letter expression for the best achievable asymptotic performance.

Our main novel messages in this work can be summarized as follows.

1. We essentially derive an asymptotic formula for the rate–distortion function of an individual sequence. It is given by the normalized negative logarithm of the universal probability of a sphere of radius nD (in the distortion metric sense) around the given source sequence.
2. We establish the universality of the LZ probability distribution, not only in the context of lossless compression, but also for lossy compression, in the role of a universal distribution for independent random selection of a rate–distortion codebook.
3. The expression of this rate–distortion function is intimately related to a converse result due to Kontoyiannis and Zhang [22] (in terms of the probability of a sphere), but it is more explicit in a certain sense, that will be discussed in Section 5.
4. We provide some intuitive insight regarding this rate–distortion function by demonstrating the analogy to the ordinary rate–distortion function (see Section 5).
5. We show that our achievability scheme outperforms the (seemingly) natural extension of LZ compression to the lossy case, where one seeks, within a sphere of radius nD, the reproduction vector whose LZ code length is minimal (see Section 5).

The outline of this paper is as follows. In Section 2, we establish the notation conventions, define a few terms and quantities, and provide some background. In Section 3, we present the converse theorem and its proof. In Section 4, we present the achievability theorem and prove it. Finally, in Section 5, we summarize the paper and discuss our results.

## 2. Notation, Definitions, and Background

Throughout the paper, random variables will be denoted by capital letters, specific values they may take will be denoted by the corresponding lower case letters, and their alphabets will be denoted by calligraphic letters. Random vectors and their realizations will be denoted, respectively, by capital letters and the corresponding lower case letters, both in a bold face font. Their alphabets will be superscripted by their dimensions. The source vector of length *n*, $\left(x_{1},\ldots ,x_{n}\right)$, with components, $x_{i}$, i=1,…,n, from a finite alphabet, X, will be denoted by x. The set of all such *n*-vectors will be denoted by $X^{n}$, which is the *n*th-order Cartesian power of X. Likewise, a reproduction vector of length *n*, $\left({\hat{x}}_{1},\ldots ,{\hat{x}}_{n}\right)$, with components, ${\hat{x}}_{i}$, i=1,…,n, from a finite alphabet, $\hat{X}$, will be denoted by $\hat{x}\in {\hat{X}}^{n}$. We denote the cardinalities of X and $\hat{X}$ by *J* and *K*, respectively.

For i≤j, the notation $x_{i}^{j}$ will be used to denote the substring $\left(x_{i},x_{i+1},\ldots ,x_{j}\right)$. Probability distributions will be denoted by the letter *P* or *Q* with possible subscripts, depending on the context. The probability of an event E will be denoted by Pr{E}, and the expectation operator with respect to a probability distribution *P* will be denoted by E{·}. For two positive sequences, $a_{n}$ and $b_{n}$, the notation $a_{n}\overset{\cdot }{=}b_{n}$ will stand for equality in the exponential scale, that is, $\lim_{n\to \infty }\frac{1}{n}\log\frac{a_{n}}{b_{n}}=0$. Similarly, $a_{n}\overset{\cdot }{\leq }b_{n}$ means that ${lim sup}_{n\to \infty }\frac{1}{n}\log\frac{a_{n}}{b_{n}}\leq 0$, and so on. The indicator function of an event E will be denoted by I{E}. The notation ${\left[x\right]}_{+}$ will stand for max{0,x}. The logarithmic function, logx, will be understood to be defined to the base 2. Logarithms to the base *e* will be denoted by ln.

Let *ℓ* be a positive integer that divides *n*. The *ℓ*th-order empirical distribution of $x\in X^{n}$, which will be denoted by ${\hat{P}}_{x}^{\ell }$, is the vector of relative frequencies $\left\{{\hat{P}}_{x}^{\ell }\left(a^{\ell }\right),\;a^{\ell }\in X^{\ell }\right\}$, where

(1) $${\hat{P}}_{x}^{\ell }\left(a^{\ell }\right)=\frac{\ell }{n}\sum_{i=0}^{n/\ell -1}I\left\{x_{i\ell +1}^{(i+1)\ell }=a^{\ell }\right\}.$$

The set of all *ℓ*th-order empirical distributions of sequences in $X^{n}$ will be denoted by $P_{n}^{\ell }$. For $P^{\ell }\in P_{n}^{\ell }$, the type class $\left\{x\in X^{n}:\;{\hat{P}}_{x}^{\ell }=P^{\ell }\right\}$ will be denoted by $T_{n}\left(P^{\ell }\right)$. Likewise, $T_{n}\left(Q^{\ell }\right)$ will denote $\left\{\hat{x}\in {\hat{X}}^{n}:\;{\hat{P}}_{\hat{x}}^{\ell }=Q^{\ell }\right\}$, where ${\hat{P}}_{\hat{x}}^{\ell }$ is the *ℓ*th-order empirical distribution of $\hat{x}$. Finally, ${\hat{P}}_{x\hat{x}}^{\ell }$ will denote the *ℓ*th-order joint empirical distribution of $\left(x,\hat{x}\right)$, i.e.,

(2) $${\hat{P}}_{x\hat{x}}^{\ell }\left(a^{\ell },b^{\ell }\right)=\frac{\ell }{n}\sum_{i=0}^{n/\ell -1}I\left\{x_{i\ell +1}^{(i+1)\ell }=a^{\ell },{\hat{x}}_{i\ell +1}^{(i+1)\ell }=b^{\ell }\right\},\;\;\;\left(a^{\ell },b^{\ell }\right)\in X^{\ell }\times {\hat{X}}^{\ell }.$$

For a given positive integer *n*, a distortion function, *d*, is a function from $X^{n}\times {\hat{X}}^{n}$ into $R^{+}$. In the two main parts of this paper, different assumptions will be imposed on the distortion function.

1. For the achievability theorem, the distortion function can be completely arbitrary.
2. For the converse theorem, we assume that $d(x,\hat{x})$ depends on x and $\hat{x}$ only via their first-order joint empirical distribution, ${\hat{P}}_{x\hat{x}}^{1}$, and that for a given such distribution, it grows linearly in *n*, that is, $d\left(x,\hat{x}\right)=n\rho \left({\hat{P}}_{x\hat{x}}^{1}\right)$, where the function ρ is independent of *n*.

Regarding item 2, additive distortion measures, which obviously comply with the requirement, are given by linear functionals of ${\hat{P}}_{x\hat{x}}^{1}$. However, here arbitrary non-linear functionals are allowed as well.

A rate–distortion block code of length *n* is a mapping, $\phi_{n}:X^{n}\to B_{n}$, $B_{n}\subset {\left\{0,1\right\}}^{*}$, that maps the space of source vectors of length *n*, $X^{n}$, into a set, $B_{n}$, of variable-length compressed bit strings. The decoder is a mapping $\psi_{n}:B^{n}\to C_{n}\subseteq {\hat{X}}^{n}$ that maps the set of compressed variable-length binary strings into a reproduction codebook $C^{n}$. A block code is called *d*-semifaithful if for every $x\in X^{n}$, $d(x,\psi_{n}\left(\phi_{n}\left(x\right)\right))\leq nD$. The code length for x, denoted L(x), is the number of bits of $\phi_{n}\left(x\right)$. Since L(x) depends on x only via $\phi_{n}\left(x\right)$, we will also denote it sometimes as $L(\phi_{n}\left(x\right))$ or by $L(\hat{x})$ ($\hat{x}$ being the reproduction vector pertaining to $\phi_{n}\left(x\right)$), with a slight abuse of notation. For the converse theorem, we assume that correspondence between $B_{n}$ and $C_{n}$ is one-to-one. For the achievability theorem, we consider prefix-free codes. Accordingly, the encoder can equivalently be presented as a cascade of a reproduction encoder (also known as a vector quantizer), which maps $X^{n}$ into $C_{n}$, followed by an entropy coder, which maps $C_{n}$ into $B_{n}$ with no additional loss of information.

For the purpose of presenting both the converse theorem and the achievability theorem, we need to recall a few terms and facts concerning the 1978 version of the LZ algorithm (also known as the LZ78 algorithm) [27]. The incremental parsing procedure of the LZ78 algorithm is a procedure of sequentially parsing a vector $\hat{x}\in {\hat{X}}^{n}$ such that each new phrase is the shortest string that has not been encountered before as a parsed phrase, with the possible exception of the last phrase which might be incomplete. For example, the incremental parsing of the vector $\hat{x}=abbabaabbaaabaa$ is a,b,ba,baa,bb,aa,ab,aa. Let $c(\hat{x})$ denote the number of phrases in $\hat{x}$ resulting from the incremental parsing procedure. Let $LZ(\hat{x})$ denote the length of the LZ78 binary compressed code for $\hat{x}$. According to ([27], Theorem 2),

(3) $$\begin{array}{ccc} LZ(\hat{x}) & \leq & \left[c\left(\hat{x}\right)+1\right]\log\left\{2K\left[c\left(\hat{x}\right)+1\right]\right\}\\ & = & c\left(\hat{x}\right)\log\left[c\left(\hat{x}\right)+1\right]+c\left(\hat{x}\right)\log\left(2J\right)+\log\left\{2K\left[c\left(\hat{x}\right)+1\right]\right\}\\ & = & c\left(\hat{x}\right)\log c\left(\hat{x}\right)+c\left(\hat{x}\right)\log\left[1+\frac{1}{c(\hat{x})}\right]+c\left(\hat{x}\right)\log\left(2K\right)+\log\left\{2K\left[c\left(\hat{x}\right)+1\right]\right\}\\ & \leq & c\left(\hat{x}\right)\log c\left(\hat{x}\right)+\log e+\frac{n(\log K)\log(2K)}{(1-\epsilon_{n})\log n}+\log\left[2K\left(n+1\right)\right]\\ & \overset{▵}{=} & c\left(\hat{x}\right)\log c\left(\hat{x}\right)+n\cdot \epsilon \left(n\right), \end{array}$$

where we remember that *K* is the cardinality of $\hat{X}$, and where ϵ(n) clearly tends to zero as n→∞, at the rate of 1/logn. We next define a *universal probability distribution* (see also [35,36]):

(4) $$U\left(\hat{x}\right)=\frac{2^{-LZ(\hat{x})}}{\sum_{{\hat{x}}^{\prime }\in {\hat{X}}^{n}}2^{-LZ({\hat{x}}^{\prime })}},\;\;\;\;\hat{x}\in {\hat{X}}^{n}.$$

Finally, we define the *D*-sphere around x as

(5) $$S\left(x,D\right)=\{\hat{x}:\;d\left(x,\hat{x}\right)\leq nD\},$$

and

(6) $$U\left[S\left(x,D\right)\right]=\sum_{\hat{x}\in S\left(x,D\right)}U\left(\hat{x}\right).$$

For later use, we also define

(7) $$\hat{S}\left(\hat{x},D\right)=\left\{x:\;d\left(x,\hat{x}\right)\leq nD\right\}.$$

Our purpose is to derive upper and lower bounds on the smallest achievable code length, L(x), for *d*-semifaithful block codes of length *n*, and individual sequences, {x}, from a given *ℓ*th-order-type class, $T_{n}\left(P^{\ell }\right)$. As will be seen shortly, in both the converse and the achievability theorems, the main term of the bound on the length function will be −log(U[S(x,D)]).

## 3. The Converse Theorem

The following converse theorem asserts that even if the type class of the source vector was known to the decoder ahead of time, the code length could not be much smaller than −log(U[S(x,D)]) for the vast majority of the codewords pertaining to that type.

**Theorem** **1.** *Let ℓ be a positive integer that divides n and let ${\hat{P}}^{\ell }$ be an arbitrary empirical distribution pertaining to a certain type class, $T_{n}\left({\hat{P}}^{\ell }\right)$, of source sequences in $X^{n}$. Let d be a distortion function that depends on $\left(x,\hat{x}\right)$ only via ${\hat{P}}_{x\hat{x}}^{1}$. Then, for every d-semifaithful variable-length block code, with one-to-one correspondence between $B_{n}$ and $C_{n}$, and for every ϵ>0, the following lower bound applies to a fraction of at least $\left(1-2n^{-\epsilon }\right)$ of the codewords, $\left\{\phi_{n}\left(x\right),\;x\in T_{n}\left({\hat{P}}^{\ell }\right)\right\}$:*

(8) $$L\left(\phi_{n}\left(x\right)\right)\geq -\log\left(U\left[S\left(x,D\right)\right]\right)-n\Delta_{n}\left(\ell \right)-\epsilon \log n,$$

*where $\Delta_{n}\left(\ell \right)$ has the property $\lim_{n\to \infty }\Delta_{n}\left(\ell \right)=1/\ell$.*

As a technical note, observe that $\Delta_{n}\left(\ell \right)$ can be made small when *ℓ* is chosen to be large, as $\Delta_{n}\left(\ell \right)$ behaves like 1/ℓ for fixed *ℓ* and large *n*. This suggests that the theorem is meaningful mainly when *ℓ* is appreciably large, which is not surprising, because the larger *ℓ* is, the better one can exploit empirical dependencies within the source sequence. Enlarging *ℓ* also increases the asymptotic lower bound and, thus, makes it tighter. An alternative approach would be to let $\ell =\ell_{n}$ grow with *n*, but sufficiently slowly such that $\Delta_{n}\left(\ell_{n}\right)\to 0$ as n→∞.

The remaining part of this section is devoted to the proof of Theorem 1. The proof is based on two main steps whose aim is to bypass the need for the traditional information-theoretic expression of the rate–distortion function. The first step is based on a simple counting argument, displayed in Equations (9)–(14) below, which leads to the identity (15) and, more specifically, to (16). Equation (16) is pivotal to the proof because it establishes exact equality between the sphere-covering ratio (on the left-hand side) and a quantity (on the right-hand side) that can be interpreted as one over the probability of falling into an nD-sphere around x under random coding with respect to the uniform distribution over the type class, $T_{n}\left(Q^{\ell }\right)$. The sphere-covering ratio on the left-hand side belongs to the converse, and the expression on the right-hand side is strongly related to achievability (see Theorem 2 below), as it directly supports random coding. Therefore, these identities create the basic link between the converse and the direct theorems. The second step is to replace the uniform distribution over $T_{n}\left(Q^{\ell }\right)$ by the universal LZ distribution, $U\left(x\right)\propto 2^{-LZ(\hat{x})}$, and thereby relax the dependence upon $P^{\ell }$ (or $Q^{\ell }$), as well as on the parameter *ℓ*, without sacrificing the asymptotic performance. This proof is entirely different from the one in [34].

**Proof.**  We first establish a relationship that will be used later on. For two given types $T_{n}\left(P^{\ell }\right)\subset X^{n}$ and $T_{n}\left(Q^{\ell }\right)\subset {\hat{X}}^{n}$, consider the quantity,

(9) $$N\left(D\right)=\sum_{x,\hat{x}}I\left\{x\in T_{n}\left(P^{\ell }\right),\;\hat{x}\in T_{n}\left(Q^{\ell }\right),\;d\left(x,\hat{x}\right)\leq nD\right\}.$$

We can evaluate N(D) in two ways. The first is as follows:

(10) $$\begin{array}{ccc} N(D) & = & \sum_{x\in T_{n}\left(P^{\ell }\right)}|T_{n}\left(Q^{\ell }\right)⋂S\left(x,D\right)| \end{array}$$

(11) $$\begin{array}{ccc} & = & |T_{n}\left(P^{\ell }\right)\left|\cdot \right|T_{n}\left(Q^{\ell }\right)⋂S\left(x,D\right)|, \end{array}$$

where the second equality is since $|T_{n}\left(Q^{\ell }\right)⋂S\left(x,D\right)|$ is the same for all $x\in T_{n}\left(P^{\ell }\right)$, due to the permutation-invariance assumption on the distortion function. By the same token, we can also express N(D) in the following manner:

(12) $$\begin{array}{ccc} N(D) & = & \sum_{\hat{x}\in T_{n}\left(Q^{\ell }\right)}|T_{n}\left(P^{\ell }\right)⋂\hat{S}\left(\hat{x},D\right)| \end{array}$$

(13) $$\begin{array}{ccc} & = & |T_{n}\left(Q^{\ell }\right)\left|\cdot \right|T_{n}\left(P^{\ell }\right)⋂\hat{S}\left(\hat{x},D\right)|, \end{array}$$

which follows from the same consideration by symmetry. It follows then that

(14) $$|T_{n}\left(P^{\ell }\right)\left|\cdot \right|T_{n}\left(Q^{\ell }\right)⋂S\left(x,D\right)|=\left|T_{n}\left(Q^{\ell }\right)\right|\cdot |T_{n}\left(P^{\ell }\right)⋂\hat{S}\left(\hat{x},D\right)|,$$

or, equivalently,

(15) $$\frac{|T_{n}\left(P^{\ell }\right)|}{\left|T_{n}\left(P^{\ell }\right)⋂\hat{S}\left(\hat{x},D\right)\right|}=\frac{|T_{n}\left(Q^{\ell }\right)|}{\left|T_{n}\left(Q^{\ell }\right)⋂S\left(x,D\right)\right|}.$$

Now, let $Q_{*}^{\ell }$ be the type of $\hat{x}$ that maximizes $|T_{n}\left(P^{\ell }\right)⋂\hat{S}\left(\hat{x},D\right)|$. Then, the last equation implies that

(16) $$\frac{|T_{n}\left(P^{\ell }\right)|}{\max_{\hat{x}\in {\hat{X}}^{n}}|T_{n}\left(P^{\ell }\right)⋂\hat{S}\left(\hat{x},D\right)|}=\frac{|T_{n}\left(Q_{*}^{\ell }\right)|}{\left|T_{n}\left(Q_{*}^{\ell }\right)⋂S\left(x,D\right)\right|},\;\;\;\forall \;x\in T_{n}\left(P^{\ell }\right).$$

This relationship will be used shortly.

Let $P^{\ell }\in P_{n}^{\ell }$ be given. Any *d*-semifaithful code must fully cover the type class $T_{n}\left(P^{\ell }\right)$ with spheres of radius nD (henceforth, referred to as *D*-spheres) centered at the various codewords. Let ${\hat{x}}_{1},\ldots ,{\hat{x}}_{M}\in {\hat{X}}^{n}$ be *M* codewords. The number of members of $T_{n}\left(P^{\ell }\right)$ that are covered by ${\hat{x}}_{1},\ldots ,{\hat{x}}_{M}\in {\hat{X}}^{n}$ is upper-bounded as follows.

(17) $$\begin{array}{ccc} G & = & \left|\overset{M}{\underset{i=1}{⋃}}\left[T_{n}\left(P^{\ell }\right)⋂\hat{S}\left({\hat{x}}_{i},D\right)\}\right]\right|\\ & \leq & \sum_{i=1}^{M}|T_{n}\left(P^{\ell }\right)⋂\hat{S}\left({\hat{x}}_{i},D\right)\}|\\ & \leq & M\cdot \max_{\hat{x}\in \hat{X^{n}}}|T_{n}\left(P^{\ell }\right)⋂\hat{S}\left(\hat{x},D\right)\}|, \end{array}$$

and so, the necessary condition for complete covering, which is $G\geq |T_{n}\left(P^{\ell }\right)|$, amounts to

(18) $$\begin{array}{ccc} M & \geq & \frac{|T_{n}\left(P^{\ell }\right)|}{\max_{\hat{x}\in \hat{X^{n}}}|T_{n}\left(P^{\ell }\right)⋂\hat{S}\left(\hat{x},D\right)\}|}\\ & = & \frac{|T_{n}\left(Q_{*}^{\ell }\right)|}{\left|T_{n}\left(Q_{*}^{\ell }\right)⋂S\left(x,D\right)\right|}\\ & \overset{▵}{=} & M_{0}, \end{array}$$

where the second line is from (16). Consider now a variable-length code with a codebook of size *M*. Let $L(\hat{x})$ denote the length (in bits) of the compressed binary string that represents $\hat{x}$. The number of codewords with $L(\hat{x})\leq \log M-\epsilon \log n$ is upper-bounded as follows:

(19) $$\begin{array}{ccc} \left|\{\hat{x}\in C_{n}:\;L\left(\hat{x}\right)\leq \log M-\epsilon \log n\}\right| & = & \sum_{k=1}^{\log M-\epsilon \log n}\left|\left\{\hat{x}:\;L\left(\hat{x}\right)=k\right\}\right|\\ & \leq & \sum_{k=1}^{\log M-\epsilon \log n}2^{k}\\ & = & 2^{\log M-\epsilon \log n+1}-1\\ & < & 2n^{-\epsilon }M, \end{array}$$

where in the first inequality we have used the assumed one-to-one property of the mapping between the reproduction codewords and their variable-length compressed binary representations. It follows then, that for at least $M(1-2n^{-\epsilon })$ out of the *M* codewords in $C^{n}$ (that is, the vast majority of codewords), we have

(20) $$\begin{array}{ccc} L(\phi_{n}\left(x\right)) & \geq & \log M-\epsilon \log n\\ & \geq & \log M_{0}-\epsilon \log n\\ & = & -\log\left(\frac{\left|T_{n}\left(Q_{*}^{\ell }\right)⋂S\left(x,D\right)\right|}{|T_{n}\left(Q_{*}^{\ell }\right)|}\right)-\epsilon \log n\\ & = & -\log\left[\sum_{\hat{x}\in S\left(x,D\right)}U_{Q_{*}}\left(\hat{x}\right)\right]-\epsilon \log n, \end{array}$$

where $U_{Q_{*}}$ is the uniform probability distribution across the type class $Q_{*}^{\ell }$, i.e.,

(21) $$U_{Q_{*}}\left(\hat{x}\right)=\left\{\begin{array}{cc} \frac{1}{|T_{n}\left(Q_{*}^{\ell }\right)|} & \hat{x}\in T_{n}\left(Q_{*}^{\ell }\right)\\ 0 & \mathrm{elsewhere} \end{array}\right.$$

We now argue that for every $\hat{x}\in {\hat{X}}^{n}$

(22) $$U_{Q_{*}}\left(\hat{x}\right)\leq \exp_{2}\left\{-LZ\left(\hat{x}\right)+n\Delta_{n}\left(\ell \right)\right\}.$$

For $\hat{x}\notin T_{n}\left(Q_{*}^{\ell }\right)$, this is trivial as the left-hand side is equal to zero. For $\hat{x}\in T_{n}\left(Q_{*}^{\ell }\right)$, we have the following consideration. In ([37], Equation (30)), it is shown (with somewhat different notation) that a certain quantity called the (unnormalized) *s*-state complexity [27] of $\hat{x}$, $n\rho_{s}\left(\hat{x}\right)$, for $s=S\left(\ell \right)\overset{▵}{=}\left(K^{\ell +1}-1\right)/\left(K-1\right)$, is upper-bounded by

(23) $$n\rho_{s}\left(\hat{x}\right)\leq \frac{n}{\ell }\left[H_{Q_{*}}\left({\hat{X}}^{\ell }\right)+1\right],$$

where

(24) $$H_{Q_{*}}\left({\hat{X}}^{\ell }\right)=-\sum_{b^{\ell }\in {\hat{X}}^{\ell }}Q_{*}^{\ell }\left(b^{\ell }\right)\log Q_{*}^{\ell }\left(b^{\ell }\right).$$

On the other hand, $n\rho_{s}\left(\hat{x}\right)$ is lower-bounded (see [37], Equation (32)) by

(25) $$n\rho_{s}\left(\hat{x}\right)\geq c\left(\hat{x}\right)\log c\left(\hat{x}\right)-\frac{n\log(4S^{2}\left(\ell \right))\log K}{(1-\epsilon_{n})\log n}-S^{2}\left(\ell \right)\log\left(4S^{2}\left(\ell \right)\right),$$

where $\epsilon_{n}\to 0$, and so,

(26) $$\frac{n}{\ell }\left[H_{Q_{*}}\left({\hat{X}}^{\ell }\right)+1\right]\geq c\left(\hat{x}\right)\log c\left(\hat{x}\right)-\frac{n\log(4S^{2}\left(\ell \right))\log K}{(1-\epsilon_{n})\log n}-S^{2}\left(\ell \right)\log\left(4S^{2}\left(\ell \right)\right).$$

Combining this with the inequality ([38], p. 17, Lemma 2.3),

(27) $$|T_{n}\left(Q_{*}^{\ell }\right)|\geq {\left(\frac{n}{\ell }+1\right)}^{-K^{\ell }}\cdot 2^{nH_{Q_{*}}\left({\hat{X}}^{\ell }\right)/\ell },$$

we have

(28) $$\begin{array}{ccc} \log|T_{n}\left(Q_{*}^{\ell }\right)| & \geq & c\left(\hat{x}\right)\log c\left(\hat{x}\right)-n\delta_{n}\left(\ell \right)-K^{\ell }\log\left(\frac{n}{\ell }+1\right)\\ & \geq & LZ\left(\hat{x}\right)-n\epsilon \left(n\right)-n\delta_{n}\left(\ell \right)-K^{\ell }\log\left(\frac{n}{\ell }+1\right)\\ & \overset{▵}{=} & LZ\left(\hat{x}\right)-n\Delta_{n}\left(\ell \right), \end{array}$$

where

(29) $$\delta_{n}\left(\ell \right)=\frac{\log[4S^{2}\left(\ell \right)]\log K}{(1-\epsilon_{n})\log n}+\frac{S^{2}\left(\ell \right)\log\left[4S^{2}\left(\ell \right)\right]}{n}+\frac{1}{\ell },$$

and where the second inequality in (28) follows from (3). The last line of (28) is equivalent to (22). It follows then, that for at least $M(1-2\cdot n^{-\epsilon })$ out of the *M* codewords in $C^{n}$,

(30) $$\begin{array}{ccc} L(\phi_{n}\left(x\right)) & \geq & -\log\left[\sum_{\hat{x}\in S\left(x,D\right)}2^{-LZ(\hat{x})}\right]-n\Delta_{n}\left(\ell \right)-\epsilon \log n\\ & = & -\log\left[\sum_{\hat{x}\in S\left(x,D\right)}\frac{2^{-LZ(\hat{x})}}{\sum_{{\hat{x}}^{\prime }\in {\hat{X}}^{n}}2^{-LZ({\hat{x}}^{\prime })}}\right]-\\ & & \log\left(\sum_{\hat{x}\in {\hat{X}}^{n}}2^{-LZ(\hat{x})}\right)-n\Delta_{n}\left(\ell \right)-\epsilon \log n\\ & \geq & -\log\left(U\left[S\left(x,D\right)\right]\right)-n\Delta_{n}\left(\ell \right)-\epsilon \log n, \end{array}$$

where in the last step we have applied Kraft’s inequality to the LZ code-length function. This completes the proof of Theorem 1.   □

## 4. The Achievability Theorem

The lower bound of Theorem 1 naturally suggests achievability using the universal distribution, *U*, for random selection of the various codewords. The basic idea is quite standard and simple: the quantity U[S(x,D)] is the probability that a single randomly chosen reproduction vector, drawn under *U*, would fall within distance nD from the source vector, x. If all reproduction vectors are drawn independently under *U*, then the typical number of such random selections that it takes before one sees the first one in S(x,D) is of the exponential order of 1/U[S(x,D)]. Given that the codebook is revealed to both the encoder and decoder, once it has been selected, the encoder merely needs to transmit the index of the first such reproduction vector within the codebook, and the description length of that index can be made essentially as small as log{1/U[S(x,D)]}=−log(U[S(x,D)]). We use this simple idea to prove achievability for an arbitrary distortion measure. The proof is similar to the parallel proof in [34], and it is presented here mainly for completeness.

The achievability theorem is the following.

**Theorem** **2.** *Let $d:X^{n}\times {\hat{X}}^{n}\to R^{+}$ be an arbitrary distortion function. Then, for every ϵ>0, there exists a sequence of d-semifaithful, variable-length block codes of block length n, such that for every $x\in X^{n}$, the code length for x is upper-bounded by*

(31) $$L\left(x\right)\leq -\log\left(U\left[S\left(x,D\right)\right]\right)+\left(2+\epsilon \right)\log n+c+\delta_{n},$$

*where c>0 is a constant and $\delta_{n}=O\left(nJ^{n}e^{-n^{1+\epsilon }}\right)$.*

**Proof.**  The proof is based on the following simple well-known fact: Given a source vector $x\in X^{n}$ and a codebook, $C_{n}$, let I(x) denote the index, *i*, of the first vector, ${\hat{x}}_{i}$, such that $d(x,{\hat{x}}_{i})\leq nD$, namely, ${\hat{x}}_{i}\in S\left(x,D\right)$. If all reproduction vectors are drawn independently under *U*, then, for every positive integer, *N*:

(32) $$Pr\left\{I\left(x\right)>N\right\}={\left(1-U\left[S\left(x,D\right)\right]\right)}^{N}=\exp\{N\ln\left(1-U\left[S\left(x,D\right)\right]\right\}\leq \exp\left\{-N\cdot U\left[S\left(x,D\right)\right]\right\},$$

and so, if $N=N_{n}=e^{\lambda_{n}}/U\left[S\left(x,D\right)\right]$, for some arbitrary positive sequence, $\left\{\lambda_{n}\right\}$, that tends to infinity, then

(33) $$Pr\left\{I\left(x\right)>N_{n}\right\}\leq \exp\left\{-e^{\lambda_{n}}\right\}.$$

This fact will be used few times in this section.   □

For later use, we also need the following uniform lower bound to U[S(x,D)]: for a given x, let ${\hat{x}}_{0}\in {\hat{X}}^{n}$ denote an arbitrary reproduction vector within S(x,D). Then,

(34)U[S(x,D)]≥U(x^0)(35)=2−LZ(x^0)∑x^∈X^n2−LZ(x^)(36)≥2−LZ(x^0).

Next, observe that $LZ({\hat{x}}_{0})$ is maximized by the *K*-ary extension of the counting sequence ([27], p. 532), which is defined as follows: for i=1,2,…,m (*m* – positive integer), let u(i) denote the *K*-ary string of length $iK^{i}$ that lists, say, in lexicographic order, all the $K^{i}$ words from ${\hat{X}}^{i}$, and let ${\hat{x}}_{0}=\left(u\left(1\right)u\left(2\right)\ldots u\left(m\right)\right)$, whose length is

(37) $$\begin{array}{ccc} n & = & \sum_{i=1}^{m}iK^{i}\\ & = & K\cdot \sum_{i=1}^{m}iK^{i-1}\\ & = & K\cdot \frac{\partial }{\partial K}\left(\sum_{i=1}^{m}K^{i}\right)\\ & = & K\cdot \frac{\partial }{\partial K}\left(\frac{K^{m+1}-K}{K-1}\right)\\ & = & \frac{K}{{\left(K-1\right)}^{2}}\left[mK^{m+1}-\left(m+1\right)K^{m}+1\right]. \end{array}$$

The LZ incremental parsing of ${\hat{x}}_{0}$, which is exactly (u(1),u(2),…,u(m)), yields

(38) $$c\left({\hat{x}}_{0}\right)=\sum_{i=1}^{m}K^{i}=\frac{K^{m+1}-K}{K-1},$$

and so, considering Equation (3), it follows that $LZ\left({\hat{x}}_{0}\right)\leq \left(1+\epsilon_{n}\right)n\log K$ for some $\epsilon_{n}\to 0$ as n→∞. It follows then, that

(39) $$U\left[S\left(x,D\right)\right]\geq 2^{-n(1+\epsilon_{n})\log K}.$$

**Remark** **1.** *For an alternative to this upper bound on the LZ code length, one can slightly modify the LZ algorithm as follows: if $LZ(\hat{x})\leq n\log K$, use the LZ algorithm as usual, otherwise, send $\hat{x}$ uncompressed using nlogK bits. To distinguish between the two modes of operation, append a flag bit to indicate whether or not the data are LZ-compressed. The modified code length would then be $LZ^{\prime }\left(\hat{x}\right)=\min\left\{LZ\left(\hat{x}\right),n\log K\right\}+1$. Now, replace $LZ(\hat{x})$ by $LZ^{\prime }\left(\hat{x}\right)$ in all places throughout this paper, including the definition of U.*

Consider now an independent random selection of all reproduction vectors to form a codebook, $C_{n}$, of size $M=A^{n}$ (A>K) codewords, ${\hat{x}}_{1},{\hat{x}}_{2},\ldots ,{\hat{x}}_{M}$, according to *U*. Once the codebook $C_{n}=\left\{{\hat{x}}_{1},{\hat{x}}_{2},\ldots ,{\hat{x}}_{M}\right\}$ has been drawn, it is revealed to both the encoder and the decoder. Consider next the following encoder. As defined before, let I(x) be defined as the index of the first codeword that falls within S(x,D), but now, with the small modification that if none of the $A^{n}$ codewords fall in S(x,D), then we define $I\left(x\right)=A^{n}$ anyway (and then the encoding fails). Next, we define the following probability distribution over the positive integers, $1,2,\ldots ,A^{n}$:

(40) $$u\left[i\right]=\frac{1/i}{\sum_{k=1}^{A^{n}}1/k},\;\;\;\;i=1,2,\ldots ,A^{n}.$$

Given x, the encoder finds I(x) and encodes it using a variable-rate lossless code with the length function (in bits, and ignoring the integer length constraint)

(41) $$\begin{array}{ccc} L(x) & = & -\log u[I(x)]\\ & \leq & \log I\left(x\right)+\log\left(\sum_{k=1}^{A^{n}}\frac{1}{k}\right)\\ & \leq & \log I\left(x\right)+\log\left(\ln A^{n}+1\right)\\ & = & \log I(x)+\log(n\ln A+1)\\ & \leq & \log I(x)+\log n+c, \end{array}$$

where c=log(lnA+1). It follows that the expected codeword length for $x\in X^{n}$ (with respect to the randomness of the code) is upper-bounded by

(42) $$\begin{array}{ccc} E\{L(x)\} & \leq & E\{\log I(x)\}+\log n+c\\ & \leq & \log E\{I(x)\}+\log n+c\\ & = & \log\left(\sum_{k=1}^{A^{n}}k\cdot {\left(1-U[S(x,D)]\right)}^{k-1}\cdot U\left[S\left(x,D\right)\right]+A^{n}\cdot {\left(1-U[S(x,D)]\right)}^{A^{n}}\right)+\log n+c\\ & = & \log\left(\sum_{k=1}^{\infty }\min\left\{k,A^{n}\right\}\cdot {\left(1-U[S(x,D)]\right)}^{k-1}\cdot U\left[S\left(x,D\right)\right]\right)+\log n+c\\ & \leq & \log\left\{\sum_{k=1}^{\infty }k\cdot {\left(1-U[S(x,D)]\right)}^{k-1}\cdot U\left[S\left(x,D\right)\right]\right\}+\log n+c\\ & = & \log\left(\frac{1}{U[S(x,D)]}\right)+\log n+c, \end{array}$$

and we denote

(43) $$L^{+}\left(x\right)\overset{▵}{=}\log\left(\frac{1}{U[S(x,D)]}\right)+\log n+c.$$

Consider now the quantity

(44) $$\begin{array}{ccc} E_{n} & \overset{▵}{=} & E\{\max(\max_{x\in X^{n}}I\left\{d\left(x,\hat{X}\right)>nD\right\},\\ & & {\left[\max_{x\in X^{n}}\left(L\left(x\right)-L^{+}\left(x\right)-\left(1+\epsilon \right)\log n\right)\right]}_{+})\}, \end{array}$$

where the expectation is with respect to the randomness of the code $C_{n}$. If $E_{n}$ can be upper-bounded by $\delta_{n}$, which tends to zero as n→∞, this will imply that there must exist a code for which both

(45) $$\max_{x\in X^{n}}I\left\{d\left(x,\hat{x}\right)>nD\right\}\leq \delta_{n}$$

and

(46) $$\max_{x\in X^{n}}\left(L\left(x\right)-L^{+}\left(x\right)-\left(1+\epsilon \right)\log n\right)\leq \delta_{n}$$

at the same time. Observe that since the left-hand side of (45) is either zero or one, then if we know that it must be less than $\delta_{n}\to 0$ for some codebook $C_{n}$, it means that it must vanish as soon as *n* is large enough such that $\delta_{n}<1$, namely, $d(x,\hat{x})\leq nD$ for all x, in other words, the code is *d*-semifaithful. Furthermore, by (46), for the same codebook, we must have

(47) $$L\left(x\right)\leq L^{+}\left(x\right)+\left(1+\epsilon \right)\log n+\delta_{n}\;\;\;\;x\in X^{n},$$

and $\delta_{n}$ adds a negligible redundancy term.

To prove that $E_{n}\to 0$, we first use the simple fact that the maximum of two non-negative numbers is upper-bounded by their sum, i.e.,

(48) $$\begin{array}{ccc} E_{n} & \leq & E\left\{\max_{x\in X^{n}}I\left\{d\left(x,\hat{X}\right)>nD\right\}\right\}+\\ & & E\left\{{\left[\max_{x\in X^{n}}\left(L\left(x\right)-L^{+}\left(x\right)-\left(1+\epsilon \right)\log n)\right)\right]}_{+}\right\}, \end{array}$$

and, therefore, it is sufficient to prove that each one of these terms tends to zero. As for the first term, we have:

(49) $$\begin{array}{ccc} E\left\{\max_{x\in X^{n}}I\left\{d\left(x,\hat{X}\right)>nD\right\}\right\} & \leq & E\left\{\sum_{x\in X^{n}}I\left\{d\left(x,\hat{X}\right)>nD\right\}\right\}\\ & = & \sum_{x\in X^{n}}E\left\{I\{d\left(x,\hat{X}\right)>nD\}\right\}\\ & = & \sum_{x\in X^{n}}Pr\left\{d\left(x,\hat{X}\right)>nD\right\}\\ & = & \sum_{x\in X^{n}}{\left(1-U[S(x,D)]\right)}^{A^{n}}\\ & \leq & \sum_{x\in X^{n}}\exp\left\{-A^{n}U\left[S\left(x,D\right)\right]\right\}\\ & \overset{\left(a\right)}{\leq } & \sum_{x\in X_{n}}\exp\left\{-\exp\left\{n\left[\ln A-(1+\epsilon_{n})\ln K\right]\right\}\right\}\\ & \leq & J^{n}\exp\left(-\exp\left\{n\left[\ln A-(1+\epsilon_{n})\ln K\right]\right\}\right\}, \end{array}$$

where in (a) we have used (39). This quantity decays double-exponentially rapidly as n→∞ since we have assumed A>K.

As for the second term of (48), we have:

(50) $$\begin{array}{ccc} & & E\left\{{\left[\max_{x\in X^{n}}\left(L\left(x\right)-L^{+}\left(x\right)-\left(1+\epsilon \right)\log n\right)\right]}_{+}\right\}\\ & \overset{\left(a\right)}{\leq } & E\left\{{\left[\max_{x\in X^{n}}\left(\log I\left(x\right)-\log\frac{1}{U[S(x,D)]}-\left(1+\epsilon \right)\log n\right)\right]}_{+}\right\}\\ & = & \int_{0}^{\infty }Pr\left\{\max_{x\in X^{n}}\left[\log I\left(x\right)-\log\frac{1}{U[S(x,D)]}-\left(1+\epsilon \right)\log n)\right]\geq s\right\}ds\\ & = & \int_{0}^{n\log A}Pr\left\{\max_{x\in X^{n}}\left[\log I\left(x\right)-\log\frac{1}{U[S(x,D)]}-\left(1+\epsilon \right)\log n\right]\geq s\right\}ds\\ & = & \int_{0}^{n\log A}Pr\left[\underset{x\in X^{n}}{⋃}\left\{I\left(x\right)\geq \frac{2^{(1+\epsilon )\log n+s}}{U[S(x,D)]}\right\}\right]ds\\ & \leq & \sum_{x\in X^{n}}\int_{0}^{n\log A}Pr\left\{I\left(x\right)\geq \frac{2^{(1+\epsilon )\log n+s}}{U[S(x,D)]}\right\}ds\\ & \overset{\left(b\right)}{\leq } & \sum_{x\in X^{n}}\int_{0}^{n\log A}\exp\left\{-2^{s}n^{1+\epsilon }\right\}ds\\ & \leq & J^{n}\cdot \left(n\log A\right)\cdot \exp\left\{-n^{1+\epsilon }\right\}, \end{array}$$

where in (a) we have used (41) and (43), and in (b) we have used (33). The right-most side of this chain of inequalities clearly decays as well when *n* grows without bound. This completes the proof.

## 5. Summary and Discussion

By deriving asymptotically matching upper and lower bounds, we have established the quantity $-\frac{1}{n}\log\left(U\left[S\left(x,D\right)\right]\right)$ as having the significance of an empirical rate–distortion function for individual sequences. While this quantity is not easy to calculate for large *n*, the operative meaning of our results is that we propose a universal ensemble for rate–distortion coding. According to this ensemble, the codewords are drawn independently under the probability distribution that is proportional to $2^{-LZ(\hat{x})}$.

There are several observations, insights, and perspectives that should be addressed.

*Relation to earlier converse bounds*. The converse bound is given in terms of the probability of a sphere of radius nD around the source vector x, under the universal distribution, *U*, defined in (4). This is intimately related to a converse result due to Kontoyiannis and Zhang ([22], Theorem 1, part i), which states that for any *d*-semifaithful code, there exists a probability distribution *Q* on ${\hat{X}}^{n}$ such that L(x)≥−log(Q[S(x,D)]) for all x (see also [21]). Here, upon giving up any claims on a minority of the codewords pertaining to a given type class, we derived a lower bound of essentially the same form with the benefit of specifying a concrete choice of the distribution *Q*, i.e., we propose Q=U, the universal distribution (unlike the distribution in [22] (Section III.A), which is proportional to $2^{-L(\hat{x})}$ across the codebook).

*Interpretation of the main term of the bound.* First, observe that in the special case of lossless compression (D=0), the expression −log(U[S(x,D)]) boils down to LZ(x), as expected. In the lossy case, since $LZ(\hat{x})$ is essentially bounded by a linear function of *n* (see (39)), we can approximate the main term as follows:

(51) $$\begin{array}{ccc} -\log(U[S(x,D)]) & \leq & -\log\left(\sum_{\hat{x}\in S\left(x,D\right)}2^{-LZ(\hat{x})}\right)\\ & = & -\log\left(\sum_{L\geq 1}2^{-L}\cdot |\left\{\hat{x}:\;LZ\left(\hat{x}\right)=L\right\}⋂S\left(x,D\right)|\right)\\ & \approx & \min_{L\geq 1}\left\{L-\log|\left\{\hat{x}:\;LZ\left(\hat{x}\right)=L\right\}⋂S\left(x,D\right)|\right\}. \end{array}$$

This expression, when normalized by *n*, can be viewed as a certain extension of the rate–distortion function, from the memoryless case to the general case, in the following sense. For a memoryless source *P*, the rate–distortion function has the following representation, which is parallel to (51):

(52) $$R\left(D\right)=\min_{P_{\hat{X}}}\left[H\left(\hat{X}\right)-\max_{\left\{P_{X|\hat{X}}:\;Ed\left(X,\hat{X}\right)\leq D,\;P_{X}=P\right\}}H\left(\hat{X}|X\right)\right],$$

where the maximum over the empty set is understood to be −∞. Indeed, if we replace *U* by the the uniform distribution across the first-order type pertaining to the optimal $P_{\hat{X}}$, this is the corresponding single-letter expression of $-\log(P_{\hat{X}}\left[S\left(x,D\right)\right])$ that is obtained using the method in [38].

*Comparing to the LZ description length of the most compressible $\hat{x}\in S\left(x,D\right)$.* Since our achievable bound involves LZ compression, it is interesting to compare it to the conceptually simple coding scheme that encodes x by the vector $\hat{x}$ that minimizes $LZ(\hat{x})$ within S(x,D). Consider the following chain of equalities and inequalities:

(53) $$\begin{array}{ccc} \min_{\hat{x}\in S\left(x,D\right)}LZ\left(\hat{x}\right) & = & -\log\left(\max_{\hat{x}\in S\left(x,D\right)}2^{-LZ(\hat{x})}\right)\\ & \geq & -\log\left(\sum_{\hat{x}\in S\left(x,D\right)}2^{-LZ(\hat{x})}\right)\\ & \geq & -\log\left(\sum_{\hat{x}\in S\left(x,D\right)}\frac{2^{-LZ(\hat{x})}}{\sum_{{\hat{x}}^{\prime }\in {\hat{X}}^{n}}2^{-LZ({\hat{x}}^{\prime })}}\right)\\ & = & -\log(U[S(x,D)]), \end{array}$$

which means that the performance of our proposed scheme is never worse (and conceivably often much better) than that of selecting the vector $\hat{x}$ with the smallest $LZ(\hat{x})$ among all reproduction vectors in S(x,D). The reason for the superiority of the proposed scheme is that it takes advantage of the fact that $\hat{x}$ cannot be any vector in ${\hat{X}}^{n}$, but it must be a member of the codebook, $C_{n}$, i.e., one of the possible outputs of a vector quantizer. On the other hand, in view of [27], $\min_{\hat{x}\in S\left(x,D\right)}LZ\left(\hat{x}\right)$ is essentially achievable upon compressing the output of a certain reproduction encoder (or vector quantizer) using a finite-state encoder, but a finite-state machine does not have enough memory resources to take advantage of the fact that vectors outside $C_{n}$ cannot be encountered by the encoder. Another interesting comparison between the two schemes is in terms of computational complexity. While in our scheme, the encoder has to carry out typically about 1/U[S(x,D)] distortion calculations before finding the first $\hat{x}\in S\left(x,D\right)$, in the alternative scheme the number of calculations is |S(x,D)|. The former is a decreasing function of *D*, whereas the latter is an increasing function of *D*. Therefore, in terms of computational complexity, the preference between the two schemes might depend on *D*. Specifically, for an additive distortion measure, it is easy to see that

(54) $$\frac{1}{U[S(x,D)]}\overset{\cdot }{\leq }\exp_{2}\left\{nR\left(D,P_{x}^{1}\right)\right\}$$

and, by the method of types [38]:

(55) $$\left|S\left(x,D\right)\right|\overset{\cdot }{=}\exp_{2}\left\{nE\left(D,P_{x}^{1}\right)\right)\}\overset{▵}{=}\exp_{2}\left[\max\left\{H\left(\hat{X}|X\right),\;Ed\left(X,\hat{X}\right)\leq D,\;P_{X}=P_{x}^{1}\right\}\right].$$

Therefore, whenever *D* is large enough such that $R\left(D,P_{x}^{1}\right)<E\left(D,P_{x}^{1}\right))$, it is guaranteed that the coding scheme proposed here is computationally less demanding than the alternative scheme of minimizing $LZ(\hat{x})$ across S(x,D).

*Implementation of the random coding distribution.* The universal random coding distribution is not difficult to implement. One way to achieve this is by feeding the LZ decoder with a sequence of purely random bits (fair coin tosses) until we have obtained *n* symbols at the decoder output. The details can be found in [36].

*Universality with respect to the distortion measure.* As mentioned in the Introduction, in [23,24,34], there are results on the existence of rate–distortion codes that are universal, not only in terms of the source, but also in the sense of the distortion measure. Since the proof of our achievability scheme is very similar to that of [34], it is possible to extend the achievability proof here too, so as to make our code distortion-universal for a wide class of distortion measures. This can be carried out by redefining $E_{n}$ to include the maximization of both terms over a dense grid of distortion functions, as was performed in [34]. We opted not to include this in the present paper since it is straightforward given the results we already have here and in [34].

## Data Availability

Not applicable.
